# MRI and biopsy in prostate cancer are the hot topic in this number of International Brazilian Journal of Urology

**DOI:** 10.1590/S1677-5538.IBJU.2024.03.01

**Published:** 2024-06-05

**Affiliations:** 1 Universidade do Estado do Rio de Janeiro Unidade de Pesquisa Urogenital Rio de Janeiro RJ Brasil Unidade de Pesquisa Urogenital - Universidade do Estado do Rio de Janeiro - Uerj, Rio de Janeiro, RJ, Brasil; 2 Hospital Federal da Lagoa Rio de Janeiro RJ Brasil Serviço de Urologia, Hospital Federal da Lagoa, Rio de Janeiro, RJ, Brasil

The May-June number of Int Braz J Urol presents original contributions with a lot of interesting papers in different fields: Robotic Surgery, Prostate Cancer, Infertility, Prostatic Biopsy, MRI in Prostate Cancer, Renal Cancer, Covid-19, Endourology, BPH and Urethral stricture. The papers came from many different countries such as Brazil, Italy, Canada, Spain, Japan and USA, and as usual the editor's comment highlights some of them. The editor in chief would like to highlight the following works:

Dr. Andrade and collegues from Brazil, presented in page 237 (1) a nice systematic review about the outcomes of ablative therapy and radical treatment for prostate cancer and concluded that the biochemical recurrence and urinary continence outcomes of ablative therapy and radical treatment were similar. Ablative therapy appears to have a high rate of sexual potency.

Dr. Lepine and collegues from Brazil, presented in page 250 (2) we can obseve an important sytematic review about the intraoperative Computed Tomography (ICT) for detection of residual stones in endourology procedures and concluded that the use of ICT scans during PCNL significantly increases success rates when compared to the standard fluoroscopy-guided detection of residual stones. Our findings also indicate decreased reintervention rates, with no statistically significant differences in complication rates.

Dr. Gomes and collegues from Brazil performed in page 287 (3) an interesting study about theurinary tract symptoms in a prospective cohort of COVID-19 survivors and concluded that LUTS are highly prevalent and bothersome six months after hospitalization due to COVID-19. Assessment of LUTS may help ensure appropriate diagnosis and treatment in these patients.

Dr. Paesano and collegues from Spain, performed in page 296 (4) a nice study about the effectiveness of mapping-targeted biopsies on the index lesion in transperineal prostate biopsies and concluded that this model had the potential to avoid 23.3% of prostate biopsies without missing additional csPCa cases. Mapping-targeted biopsies of the index lesion was highly effective for identifying clinically significant prostate cancer (csPCa) in fusion transperineal prostate biopsies. A developed predictive model successfully reduced the need for almost one quarter of biopsies without missing csPCa cases.

Dr. Manfredi and collegues from Italy performed in page 309 (5) a study about the new technologies in BPH treatment. The authors shows the long term functional outcomes and surgical retreatment after thulium laser enucleation of prostate and concluded that ThuLEP is associated with optimal functional outcomes and a low frequency of BPH surgical retreatment in the long-term. Baseline PV and time from surgery were pre- dictors of BPH reoperation.

Dr. Kaneko and collegues from USA and Japan performed in page 319 (6) the paper that is the cover of the present edition. This Interesting paper is about a nomogram to predict the absence of clinically significant prostate cancer in males with negative MRI and concluded that this nomogram facilitates evaluating individual probability of clinically significant prostate cancer (CSPCa) on prostatic biopsy (PBx) in males with PIRADS 1-2 mpMRI and may be used to identify those in whom PBx may be safely avoided. Black males have increased risk of CSPCa on PBx, even in the setting of PIRADS 1-2 mpMRI.

Dr. Pires and collegues from Brazil performed in page 335 (7) a nice study about the learning curve of the urology resident for conventional radical prostatectomy (ORP) similar to that of staff initiating robot-assisted radical prostatectomy (RARP) and concluded that the learning curve of RARP is equivalent to the curve of ORP. In general, the results for the robotic group were better, however, the functional results were similar between the groups, with a slight tendency of advantage for the robotic arm.

In page 261 the group of Dr. Rosito from Brazil (8) performed a interesting study about the Brazilian Portuguese validation of the patient reported outcome measure for urethral stricture surgery (USS-PROM) questionnaire and concluded that the USS-PROMbr demonstrated acceptable cross-cultural adaptation and psychometric properties, making it a valid and useful tool for evaluating patients undergoing urethroplasty.

The group of Dr. Westerman performed in page 227 (9) a nice study about the clinical safety and efficacy of microwave ablation (MWA) for small renal masses (SRMs) and concluded that these findings support the utility of CT-guided MWA as a tool for treatment of SRMs.

The Editor-in-chief expects everyone to enjoy reading.
